# The Role of Proteasome Inhibitors in Treating Acute Lymphoblastic Leukaemia

**DOI:** 10.3389/fonc.2021.802832

**Published:** 2021-12-23

**Authors:** Chun-fung Sin, Pui-hei Marcus Man

**Affiliations:** Department of Pathology, University of Hong Kong, Hong Kong, Hong Kong SAR, China

**Keywords:** proteasome inhibitors, acute lymphoblastic leukaemia, bortezomib, carfilzomib, ixazomib

## Abstract

Acute lymphoblastic leukaemia (ALL) is an aggressive haematolymphoid malignancy. The prognosis of ALL is excellent in paediatric population, however the outcome of relapse/refractory disease is dismal. Adult ALL has less favourable prognosis and relapse/refractory disease is not uncommonly encountered. Bortezomib is the first generation proteasome inhibitor licensed to treat plasma cell myeloma and mantle cell lymphoma with favourable side effect profile. Efficacy of bortezomib had been proven in other solid tumors. Clinical studies showed promising response for proteasome inhibitors in treating relapse/refractory ALL. Thus, proteasome inhibitors are attractive alternative agents for research in treating ALL. In the review article, we will introduce different proteasome inhibitors and their difference in pharmacological properties. Moreover, the mechanism of action of proteasome inhibitors on ALL will be highlighted. Finally, results of various clinical studies on proteasome inhibitors in both paediatric and adult ALL will be discussed. This review article provides the insights on the use of proteasome inhibitors in treating ALL with a summary of mechanism of action in ALL which facilitates future research on its use to improve the outcome of ALL.

## Introduction

Acute lymphoblastic leukaemia (ALL) is an aggressive haematological malignancy. The prognosis of paediatric ALL is excellent with 90% of long-term survivor (1). However, a small proportion of them still die from relapse/refractory disease. The prognosis of the adult population is poor, with only 50-60% of long-term survivor (2). The prognosis of relapse/refractory disease is dismal.

Bortezomib is approved by Food and Drug Administration (FDA) for treatment of plasma cell myeloma and mantle cell lymphoma with reasonable side effect profile (3). Since then, bortezomib is an attractive novel agent for research in the treatment of other cancers, e.g., glioblastoma multiforme and colorectal cancer (4, 5). Bortezomib is also being researched for novel treatment of ALL with significant major discoveries made. Newer proteasome inhibitors are developed with improved efficacy and side effect profile. In this review article, it summarized the differences among various proteasome inhibitors and their mechanisms of action in ALL. Recent clinical studies to evaluate the role of proteasome inhibitors in ALL were highlighted. Finally, the future prospect of research about those agents in treating ALL will be proposed.

## Differences in Pharmacological Properties of Various Proteasome Inhibitors

### Bortezomib

Bortezomib is a first generation 26S proteasome reversible inhibitor which binds to β5 subunit of chymotryptic site of 20S subunit proteasome (6). It also binds to β1 and β2 subunits at lower affinity. Bortezomib can either be administered *via* intravenous or subcutaneous route with comparable amount of systemic concentration of drug and inhibitory action of proteasome. Bortezomib has haematological toxicities including thrombocytopenia and neutropenia which are not dose-limiting (7). Other common side effects are gastrointestinal side effects including diarrhoea, nausea, and vomiting which occur in 84% of patients. The most disabling adverse effect is peripheral neuropathy which is dose limiting and route-dependent, whereas subcutaneous administration has reduced incidence of this adverse effect (6).

### Carfilzomib

Carfilzomib is a second generation proteasome inhibitor which belongs to epoxyketone group. This drug has irreversible binding towards β5 subunit with higher β5 to β2 selectivity compared with bortezomib. The binding of carfilzomib to both β5 and β2 subunit makes it effective in treating bortezomib-resistant plasma cell myeloma (6, 8). This drug can only be administrated *via* intravenous route (6).

Carfilzomib associates with less risk of peripheral neuropathy compared with bortezomib. However, carfilzomib has a higher incidence of serious cardiotoxicity (9). It could be due to reduced number of proteasome per unit of protein in cardiac muscle (6, 8). Moreover, the off-target effect of inhibiting autophagy due to activation of protein phosphatase 2A also contributes to carfilzomib-induced cardiotoxicity (10).

### Ixazomib

Ixazomib is a boronate-based third generation proteasome inhibitor licensed to treat plasma cell myeloma and it can be administrated *via* oral route. It is a prodrug which will be hydrolyzed to form active metabolite and the active metabolite is reversibly bind to β5 subunit and to lesser extent to β1 and β2 subunits of proteasome. However, the time of dissociation from β5 subunit is shorter for ixazomib when compared with bortezomib (6, 11). Because the metabolite is similar to that of bortezomib, the incidence of grade 3 or more haematological and gastrointestinal side effects is comparable with bortezomib (6). However, the risk of peripheral neuropathy is lower than that of bortezomib (6, 12, 13).

### Newer Classes of Proteasome Inhibitors

Some newer classes of proteasome inhibitors are developing, namely oprozomib, delanzomib and marizomib with more favorable side effects profile.

Delanzomib shows similar potency of proteasome inhibition with both β5 and β1 subunit binding, in contrast to bortezomib binds only β5 subunit (14). Delanzomib shows higher affinity of binding with 20 times slower rate of dissociation compared with bortezomib (15). A phase I/II study of delanzomib showed that grade 3 or above hematological side effects were common: anemia (15%), neutropenia (23%) and thrombocytopenia (54%) at a dose of 2.1 mg/m^2^. Other non-hematological side effects included rash, nausea and diarrhea. A small proportion of patients had grade 1 or 2 peripheral neuropathy (12%). No patients had grade 3 or above peripheral neuropathy (16).

Oprozomib is an epoxyketone similar to that of carfilzomib and it shows irreversible binding of β5 subunit. However, the affinity of bindings is higher than that of ixazomib (15). The most common grade 3 or above adverse events were nausea, vomiting, diarrhea and thrombocytopenia. Only rare occurrence of grade 2 or above peripheral neuropathy (17, 18).

Marizomib can bind β1, β2 and β5 subunits of 20S proteasome irreversibly with long duration (6, 19). Safety data from phase I clinical trials showed that hematological toxicities were milder than bortezomib and carfilzomib. The common non-hematological adverse events were nausea, diarrhea, fatigue. Occurrence of central nervous system toxicities including reversible hallucination and cognitive deterioration were seen. Cardiac events were much less common than bortezomib and carfilzomib. No patients had grade 3 or above peripheral neuropathy (20, 21).

## Mechanism of Action and Pre-Clinical Studies in ALL

**Figure 1:**
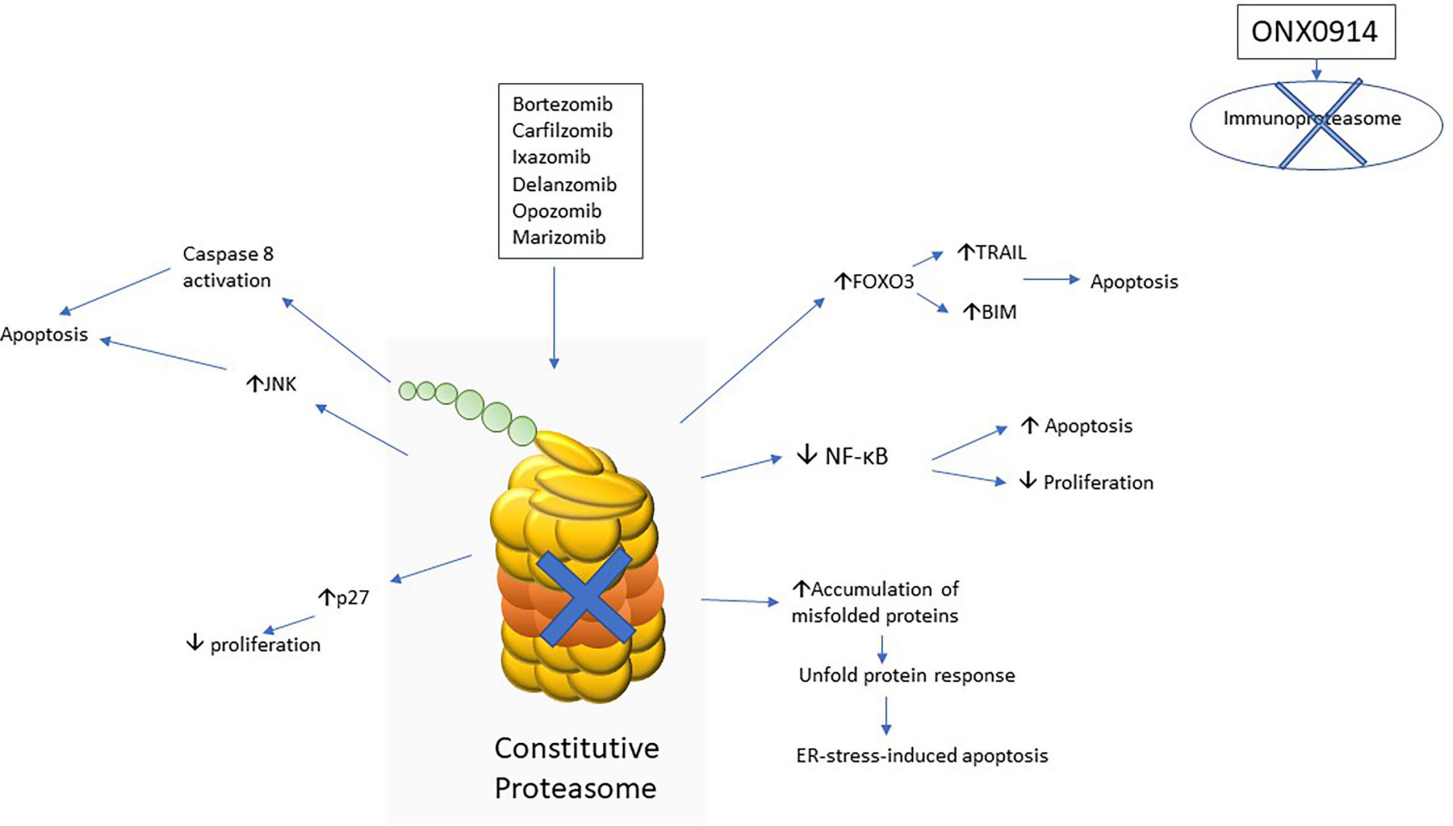
Schematic diagram to show various mechanisms of action of proteasome inhibitors implicated in acute lymphoblastic leukaemia.

The mechanism of action of proteasome inhibitors on ALL are summarized in **Figure 1**.

### NF-κB Inhibition

Study showed that bortezomib was able to stabilize IκB by preventing the degradation of IκB-α and thus inhibit NF-κB pathway in plasma cell myeloma (22). Inhibition of NF-κB pathway was found to be one of the mechanisms of therapeutic effect in ALL from a phase 1 clinical study in 2007 (23). Another earlier study in 2011 showed that bortezomib was active against relapse T lymphoblastic leukemia (T-ALL) by inhibiting the NF-κB activity in primary leukemic cells of patients (24).

*NOTCH1* activating mutation accounts for 60% of T-ALL and it drives leukemogenesis by activating NF-κB pathway (25, 26). Therefore, it is reasonable to postulate that bortezomib is effective in *NOTCH1*-mutated T-ALL. Study showed that bortezomib enhanced degradation of transcription factor Sp-1 and thus reduced *NOTCH1* transcription (27). Pre-clinical study showed that bortezomib was more effective in T-ALL than in B lymphoblastic leukaemia (B-ALL) which probably due to the action of NF-κB pathway inhibition by bortezomib (28). However, the *NOTCH1* mutation status was unknown in that study.

It is uncertain that whether the treatment of proteasome inhibitors is also effective in *NOTCH1* wild-type T-ALL, especially early T-cell precursor acute lymphoblastic leukemia (ETP-ALL) which confers a poor prognosis. More studies are needed to address this issue.

### Targeting Immunoproteasome

Immunoproteasome is a special type of proteasome that is expressed in hematopoietic cells. In contrast to the usual proteasome, or namely constitutive proteasome, immunoproteasome processes specific subunits (β1i, β2i, β5i) in the active site. This results in an increased specificity to produce peptides with hydrophobic amino acid ends, which was hypothesized to facilitate antigen presentation (29).

In a recent study on proteasome subunit expression in leukemic cells from relapsed patients, it was found that immunoproteasome was more abundantly expressed in ALL than that in acute myeloid leukemia (AML). Thus, it accounted for increased sensitivity towards multiple proteasome inhibitors, including bortezomib, carfilzomib and ONX0914 (an irreversible β5i selective inhibitor) in ALL when compared with AML (30). Targeting immunoproteasome can achieve selective toxicities towards ALL and prevent those side effects resulting from constitutive proteasome inhibition of non-lymphoid tissue, e.g., cardiac toxicities. Pre-clinical study showed that B-ALL with KMT2A-AF4 fusion and T-ALL were highly sensitive to the treatment of ONX0914 (31).

### Restoration of Forkhead Box O3 (FOXO3)

Forkhead transcription factor family (FOXO) comprises different sub-classes namely forkhead box O1 (FOXO1), forkhead box O3a (FOXO3a) and forkhead box O4 (FOXO4). This transcription factor family, FOXO is responsible for regulating gene transcription related to apoptosis and cell cycle control. The activation of Akt phosphorylates FOXO3a and leads to translocation form nucleus to cytoplasm, preventing its transcription function. Philadelphia-positive acute lymphoblastic leukemia (Ph-positive ALL) is characterized by the presence of BCR-ABL1 fusion protein resulting from translocation of chromosome 9 and 22 and PI3K-Akt pathway is constitutively activated in this subtype of B-ALL (32). Jagani, Z. et al. showed a reduced level of FOXO3a expression in a BCR-ABL1 transgenic mice model in 2009. The treatment of bortezomib restored the level of FOXO3a by preventing proteasome-mediated degradation and prolonged survival in BCR-ABL1 transgenic mice. The level of FOXO3a target proteins, namely TRAIL and BIM were increased upon bortezomib treatment resulting into apoptosis (33). These findings were also verified in patients with Ph-positive ALL. A study in 2011 showed that treatment with bortezomib induced complete hematological response with disappearance of BCR-ABL1 transcript and restoration of FOXO3a (34).

Philadelphia-like acute lymphoblastic leukemia (Ph-like ALL) is a provisional entity in World Health Organization (WHO) 2016 classification with genetic signature significantly overlapping with Ph-positive ALL and the prognosis is poor. Various kinases signaling pathway are hyperactivated, including PI3K-Akt pathway (35–37). It implies that FOXO3a will also be inactivated as a result and thus the treatment with proteasome inhibitors is potentially effective. However, no preclinical and clinical studies to verify this hypothesis.

### C-Jun Pathway

C-Jun N-terminal kinase (JNK) has three isoforms (JNK1, JNK2 and JNK3) and it belongs to mitogen activated protein kinase (MAPK). JNK is activated by MAPK kinase and JNK phosphorylates c-Jun. Activation of c-Jun will in turn activate pro-apoptotic proteins, e.g., BAD and trigger subsequent apoptosis (38). Study showed that bortezomib activates c-Jun pathway resulting into apoptosis in plasma cell myeloma (39). The c-Jun activation by bortezomib was also demonstrated in B-ALL (40). However, no studies to evaluate the effect of proteasome inhibitors on c-Jun pathway in T-ALL so far.

### Modulation of Proteostasis

Unfold protein response is triggered when there are stimuli to interfere protein folding in endoplasmic reticulum (ER) (41). If those misfolded proteins cannot be properly refolded again, they will be translocated out of ER and subjected to proteasome-mediated degradation *via* 26S subunit (42). Proteasome inhibition results into unfold protein response due to accumulation of misfolded proteins which triggered subsequent ER-stress-induced apoptosis (43). However, cells may restore proteostasis by upregulating molecular chaperones upon stimulation of unfold protein response triggered by ER stress. Therefore, maximal anti-leukemic effects can be achieved by inhibiting unfold protein response together with proteasome inhibition (44). A recent study utilized a casein kinase 2 (CK2) inhibitor, CX-4945 which inhibited the interaction between BIP (a sensor chaperone of ER stress) and Hsp90 (a molecular chaperone that stabilizes BIP). It resulted into impairment of BIP activities and thus inhibited the buffering function of unfold protein response against the ER stress induced by bortezomib pre-treatment. Similar effect was achieved by using a Hsp90 inhibitor, tanespimycin. The study also demonstrated synergistic effects of the above combination in various ALL cell lines (45).

When proteasome is inhibited, other pathways for protein haemostasis are activated to maintain cellular function which results into resistance to treatment with proteasome inhibitors. One such pathway is the activation of various molecular chaperones and deposition of misfolded protein aggregates in various deposition sites (46). Concurrent inhibition of molecular chaperones with the use of proteasome inhibitors will overcome the resistance and produce synergistic effect. Further studies on the modulation of proteostatic network by proteasome inhibitors and other chaperone inhibitors are needed in ALL.

### Other Mechanism

Study showed that bortezomib was particularly active against *KMT2A*-rearranged ALL and increased the level of both *KMT2A* and *KMT2A*-fusion proteins. Bortezomib was able to trigger apoptosis *via* caspase 8 activation and lead to BID activation, with subsequent activation of BAX and BAD. Moreover, bortezomib was able to induce G2/M cell cycle arrest *via* induction of p27 transcription through promoting transcription activity of *CDKN1B* by accumulation of *KMT2A*-fusion proteins upon bortezomib treatment (47). Similar therapeutic effect towards *KMT2A*-rearranged ALL was observed upon the treatment of carfilzomib. However, the underlying mechanism did not explore in that study (48).

## Resistance Mechanism to Proteasome Inhibitors

Multiple mechanisms which lead to resistance to proteasome inhibitors in hematological malignancies have been described (49). As β5 subunit of the proteasome is the target of most proteasome inhibitors, point mutations in the binding pocket of proteasome inhibitors are one of the mechanisms (50–52). In addition, upregulation of the β5 subunit by gene amplification leading to increased chymotrypsin activities and thus upregulation of NF-κB activities has also been suggested as a possible resistance mechanism in T-ALL cell lines (53).

Studies on reverting bortezomib resistance have been conducted. Interferon-γ (IFN-γ) is known to induce immunoproteasome expression (29). Immunoproteasomes was found to be reduced in bortezomib-resistant T-ALL cell lines. In an *in vitro* study using T-ALL cell lines, IFN-γ treated cells were able to demonstrate restored sensitivity towards proteasome inhibitors, including bortezomib, carfilzomib and ONX0914 (54). Therefore, combination of IFN-γ and bortezomib could sensitize bortezomib-resistance leukemic cells towards bortezomib.

## Clinical Studies of Various Proteasome Inhibitors

A phase 1 study in 2004 recruited 3 relapse/refractory ALL patients and treated with single agent bortezomib. The toxicities were tolerable. Those patients either showed blood count improvement or reduction in blast count, but no complete remission (CR) documented (55). Another phase 1 study in 2007 also demonstrated acceptable toxicities in pediatric patients with refractory ALL, though no clinical response observed (23) (**Table 1**).

**Table 1:** Data from clinical studies of proteasome inhibitors.

Study and year of publication	Age of patients	Number of patients recruited	Nature of disease	Brief details of study	Nature of study	Results	Remarks
Cortes et al. (2004) (55)	18-71-year-old	15 patients, including 3 ALL patients	Relapse/refractory	Determine MTD and DLTs upon single treatment of bortezomib	Phase 1 clinical trial	1. DLTs observed at 1.5mg/m^2^ including postural hypotension, GI side effects and hypokalemia 2. Four patients showed reduction in blasts count	1. Bortezomib was safe in treating adult patients with relapse/refractory acute leukaemia 2. The number of patients with ALL was too small.
Horton et al. (2007) (23)	1-18-year-old	12 patients, including 9 patients with ALL	Relapse/refractory	1. Determine MTD, DLTs upon single treatment of bortezomib 2. Determine biological effects of bortezomib	Phase 1 clinical trial	1. One patient had grade 4 neurological side effect and one patient had grade 4 febrile neutropaenia upon the dose of 1.7mg/m^2^ 2. No objective clinical response observed	1. A maximum dose of 1.7mg/m^2^ could be safely given to paediatric patients 2. Limited therapeutic efficacy of bortezomib was observed as single agent.
Messinger et al. (2010) (56)	3.3-16.4 years old	10 patients (9 patients had B-ALL, one patient had T-ALL)	Relapsed ALL	1. Combined treatment of bortezomib with dexamethasone, vincristine, doxorubicin, pegylated L-asparaginase 2. Determine safety of adding bortezomib 3. Estimate response rate of this regimen in paediatric relapse ALL	Phase 1 clinical trial	1. CR rate was 67% overall 2. Most grade 4-5 toxicities were haematological toxicities. No prolong cytopaenia observed 3. Only 2 patients had grade 1-2 peripheral neuropathy	Combined bortezomib with multidrug chemotherapy was safe in paediatric patients.
Iguchi et al. (2017) (57)	10-16 years old	6 patients with B-ALL	Refractory B-ALL	1. Determine safety of combined bortezomib and induction chemotherapy	Phase 1 clinical trial	1. Overall response rate was 80%. 2. Prolonged neutropaenia and thrombocytopaenia were observed. 3. Increased risk of severe infection 4. All had grade 1-2 peripheral neuropathy reported	1. Combined bortezomib with vincristine contributed to increasing rate of peripheral neuropathy
D. Hasegawa et al. (2019) (58)	5.8-7.3 years old	3 patients (2 had B-ALL, one had T-ALL)	Relapsed ALL	1. Determine safety and tolerability of combined bortezomib and chemotherapy	Phase 1 clinical trial	Grade 3-4 haematological side effect was reported	Combined bortezomib and chemotherapy was safe in paediatric patients
August et al. (2020) (59)	11 months – 18.5 years old	10 patients (9 had B-ALL, 1 had T-ALL)	Relapsed/refractory ALL	1. Combined treatment of bortezomib with reinduction chemotherapy 2. Determine the tolerability and response rate	Phase 1 clinical trial	1. Overall response rate was 89% 2. Two patients were MRD negative Grade 3 or above infective complications were seen in 40% of patient. 3. No report on peripheral neuropathy4 was noted.	1. Some patients with clinical response received prior allo-HSCT or CAR-T therapy. 2. The rate of infective complications was similar to those treated with chemotherapy alone.
Colunga-Pedraza et al. (2020) (60)	2-35 years old	15 patients (14 had B-ALL, 1 had T-ALL)	Relapse/refractory ALL and persistent MRD positive	1. Analysis of response rate treated with combined bortezomib and reinduction chemotherapy	Retrospective	1. Overall response rate was 60%. 2. Five patients achieved MRD negativity 3. Grade 1-2 peripheral neuropathy was reported in 33% of patient.	1. Effective and safe regimen which could be administered in outpatient setting
Yeo et al. (2016) (61)	0.75 – 23.7 years old	11 patients (10 had B-ALL, 1 had T-ALL)	Relapse/refractory ALL	1. Analysis of response rate and safety of combined bortezomib and reinduction chemotherapy	Retrospective	1. CR rate was 70% and 40% of patient achieved MRD negativity 2. Most common grade 3 or above toxicities were febrile neutropaenia, GI side effects and hyponatremia	1. Combined bortezomib and reinduction chemotherapy was effective and safe. 2. Despite 71% of patient had early relapse, CR rate was 70%.
Messinger et al. (2012) (62)	1.3 – 22.3 years old	22 patients (20 had B-ALL and 2 had T-ALL)	Relapse ALL	1. Combined treatment of bortezomib with dexamethasone, vincristine, doxorubicin, pegylated L-asparaginase 2. Determine response rate and safety profile	Phase 2 trial	1. Overall response rate was 73% 2. Grade 1-2 peripheral neuropathy was noted in 9% of patient 3. Most suffered from grade 3-4 haematological toxicities 4. Nine patients had grade 3 or above infective complications and 3 of them died.	1. B-ALL showed better response rate (80% in B-ALL vs none showed response in T-ALL) 2. High rate of infective complications was noted and administration of prophylatic antimicrobials could prevent fatal infective complications
A. Bertaina et al. (2017) (63)	2.6 – 21 years old	37 patients (30 had B-ALL and 7 had T-ALL)	Relapse/refractory ALL	1. Combined treatment of bortezomib with dexamethasone, doxorubicin, vincristine and pegylated asparaginase. 2. Assess response rate and safety	Prospective	1. Overall response rate was 73% and 38% of patient were MRD negative. 2. 2-year OS was 31.3% 3. Eight percent died from infective complications 4. Five patients had grade 3 or above peripheral neuropathy.	1. T-ALL also achieved good outcome (CR rate 71.4%). 2. Background of high risk for invasive fungal infection was noted in those patients and it contributed to the death in the trial. 3. Antimicrobial prophylaxis was needed
Horton et al. (2019) (64)	1-31 years old	135 patients (103 had B-ALL, 22 had T-ALL)	Relapse ALL	1. Combined bortezomib and reinduction chemotherapy 2. Evaluate the CR2 rate of the regimen	Phase 2 trial	1. CR2 rate was 68% for both B-ALL and T-ALL 2. MRD negativity rate after cycle 3 was 64% 3. Grade 3 neuropathy was noted in 2.8% of patient 4. Three patients died from infective complications	1. Combined bortezomib and reinduction chemotherapy was effective 2. Low rate of grade 3 or above peripheral neuropathy was observed. 3. No significant increase in fatal infective complications was noted when compared with previous cohorts
Roy et al. (2019) (65)	1-18 years old	25 patients (All patients were B-ALL)	First relapse of ALL	1. Combined treatment of bortezomib and cytarabine-based reduced intensity protocol 2. Assess response rate, EFS and OS	Phase 2 trial	1. CR2 rate was 88% 2. Post-induction MRD negative rate was 69% 3. Median period of blood count recovery was 41 days with no reports on prolonged hospital stay due to sepsis 4. One-year EFS and OS was 75% and 80% respectively	1. Combined bortezomib and reduced intensity regimen was effective in achieving CR2 and MRD negativity in B-ALL 2. Safe regimen in paediatric patients
Kaspers et al. (2018) (66)	1-18 years old	29 patients (25 had B-ALL, 4 had T-ALL)	Relapse/refractory ALL	1. Assess efficacy of combined bortezomib and less intensive reinduction chemotherapy 2. Patients were randomized into early-bortezomib group and late-bortezomib group	Open-label, randomized control phase 2 trial	1. No statistically significant difference in efficacy between early and late-bortezomib group was observed. 2. Overall response rate was 60% 3. Nine patients had febrile neutropaenia 4. Two patients had grade 3-4 peripheral neuropathy	1. Combined bortezomib with less intensive reinduction chemotherapeutic regimen was effective 2. Favorable side effect profiles with few serious adverse effects were seen.
Nachmias et al. (2018) (67)	Adults (>18 years old)	9 patients (5 had B-ALL, 4 had T-ALL)	Relapsed/refractory ALL	1. Combined treatment of bortezomib with hyper-CVAD or high-dose methotrexate and cytarabine 2. Assess response and safety	Prospective	1. Overall response rate was 78% (All patient with B-ALL and 2 patients with T-ALL showed response) 2. Only 2 patients had grade 1-2 peripheral neuropathy 3. Five patients had febrile neutropaenia but none of them had grade 3-4 sepsis	1. Combined bortezomib with chemotherapy was effective and safe in adult population. 2. Two T-ALL patients obtained CR and they were treated with 2-3 lines of therapy.
Jain et al. (2021) (68)	Age >14 years old	34 patients	Newly diagnosed Ph-ve CD20-positive B-ALL	1. Treated with combination of bortezomib, rituximab and paediatric-inspired protocol	Phase 2 clinical trial	1. Post-induction MRD negative rate was 71% (versus 52% in historical cohort) 2. Grade 3-4 infective complications was seen in 23 patients 3. One induction death versus 7.9% induction death in historical cohort 4. Grade 1-2 peripheral neuropathy was noted in 26% of patient 5. EFS and OS at 21-month was 79%.	1. Combined bortezomib, rituximab and paediatric protocol was highly effective with high MRD negativity rate 2. Low rate of severe toxicities 3. Difficult to attribute the beneficial effect on rituximab or bortezomib
Wartman t al. (2016) (69)	Adult aged >18 years old (median age 70 years old)	18 patients (17 had AML and 1 had ALL)	Relapse/refractory AML or ALL	1. To determine the MTD and DLTs of carfilzomib	Phase 1 clinical trial	1. No DLTs observed upon treatment with 56 mg/m^2^ 2. CHF exacerbation occurred in 11% of patient and they had pre-existing heart disease 3. Only modest response was observed (2 patients had partial response only)	1. Carfilzomib was safe and tolerable in adult population 2. Limited efficacy was observed upon single treatment of carfilzomib
Jonas et al. (2021) (70)	Adult aged 18-64 years old	10 patients (8 had B-ALL, 2 had T-ALL)	Newly diagnosed, deno Ph-ve ALL	1. Combined treatment of carfilzomib and hyper-CVAD 2. Determine safety and response rate	Phase 1 clinical trial	1. No patient had DLTs 2. Most grade 3-4 toxicities were haematological toxicities 3. Febrile neutropaenia occurred in 60% of patient 4. No grade 5 events and no cardiac events were noted 5. CR rate was 100% and 80% of patient were MRD negative after 4 cycles	1. High MRD negativity rate was noted when treated with this regimen 2. The regimen was safe and tolerable in adult

ALL, Acute lymphoblastic leukaemia; MTD, Maximum tolerated dose; DLTs, Dose-limiting toxicities; GI, Gastrointestinal; MRD, Minimal residual disease; B-ALL, B lymphoblastic leukaemia; CR, Complete remission; T-ALL, T lymphoblastic leukaemia; Allo-HSCT, Allogenic haemopoietic stem cell transplant; CAR-T, Chimeric antigen receptor T-cell; OS, Overall survival; EFS, Event-free survival; Ph-ve, Philadelphia chromosome negative; AML, Acute myeloid leukaemia; CHF, Congestive heart failure.

Single treatment of bortezomib had limited efficacy. Therefore, various clinical studies utilizing combined treatment strategy had been conducted. Study by Messinger et al. in 2010 recruited 10 pediatric patients with relapse/refractory ALL and treated with combination of bortezomib, dexamethasone and chemotherapy. The response rate was 67% and majority of the adverse events were grade 3-4 hematological toxicities. Only 2 patients had grade 1-2 peripheral neuropathy (56). Recent study by Iguchi et al. recruited 6 pediatric patients with relapse/refractory B-ALL and treated them with bortezomib plus induction chemotherapy. The overall response rate was 80%. However, 67% of them suffered from serious infective complications due to prolonged neutropenia. Five of them had grade 1-2 peripheral neuropathy and combination with vincristine could account for high rate of peripheral neuropathy (57). D. Hasegawa et al. recruited 3 Japanese pediatric relapse/refractory ALL patients and treated them with combined chemotherapy and bortezomib. All patients achieved CR and one patient achieved minimal residual disease (MRD) negativity after treatment. No fatal infective episodes occurred (58). Recent small-scale clinical studies also demonstrated excellent response to relapse/refractory ALL in pediatrics and young adults with CR rate around 60-80% and a substantial proportion of patients achieved MRD negativity. The side effects were acceptable (59–61).

Given the promising results of phase 1 studies, more large-scale clinical studies were conducted. Study by Messinger et al. in 2012 recruited 22 patients with relapse/refractory ALL (1-22 years old). The overall response rate was 73% with few patients developed peripheral neuropathy (62). Recent clinical studies also demonstrated excellent therapeutic efficacy and safety profile for bortezomib-based treatment. A. Bertaina et al. recruited 37 patients with relapse/refractory ALL aged 2–21-year-old. The overall response rate was 73% and 38% of them were MRD negative. Three patients died from infective complications and 5 patients had peripheral neuropathy (63). Horton *et al.* recruited 135 pediatrics and young adult patients (1-31 years old) with relapse/refractory ALL. They were treated with combined bortezomib and reinduction chemotherapy. The CR rate was 68% and 64% of patients were MRD negative after 3 cycles of treatment. The outcome of relapse/refractory T-ALL was excellent with CR rate of 68% (64). Combined bortezomib with reinduction chemotherapy also demonstrated improved event-free survival (EFS) and overall survival (OS) compared with supportive treatment in pediatric relapse/refractory ALL (65). Kaspers et al. showed 60% overall response rate in pediatric patients with relapse/refractory disease (66). Nachmias et al. showed 78% overall response rate in adult patients with relapse/refractory ALL with minimal toxicities when treated with combined chemotherapy and bortezomib (67). Jain et al. showed that combined pediatric-inspired regimen with rituximab and bortezomib was highly effective in treating newly diagnosed CD20-positive B-ALL in adolescent and adult patients. They showed that 71% of them was MRD negative after induction versus 52% when treated with chemotherapy alone (68).

Carfilzomib also showed to be active against B-ALL and T-ALL in pre-clinical studies (30, 47, 48, 71, 72). Pre-clinical study showed that ixazomib was active against ALL despite it was less potent than bortezomib (73). A phase 1 trial of carfilzomib conducted on adult patients showed acceptable side effects with only 11% of them had congestive heart failure and all of them had pre-existing heart disease (69). A recent phase 1 study recruited 10 adult patients with newly diagnosed ALL and they were treated with combined hyper-CVAD and carfilzomib. They achieved 80% of MRD negativity rate, compared with 53% when treated with hyper-CVAD alone. None of them had cardiac events (70).

The efficacy and safely of combined bortezomib and chemotherapy in treating relapse/refractory ALL had been proven in pediatric population. However, more studies are needed to evaluate this strategy in treating relapse/refractory disease in adult. The findings of high MRD negativity rate when combining proteasome inhibitors with chemotherapy in treating newly diagnosed ALL in adult is a promising finding, since MRD is crucial determinant of prognosis in adult ALL (74–76). This therapeutic strategy for newly diagnosed ALL can potentially improve outcome of ALL, particularly in adult patients.

## Concluding Landmark and Future Direction

In conclusion, the safety and efficacy of bortezomib in treating paediatric relapse/refractory ALL had been demonstrated in clinical studies. Data from clinical studies of carfilzomib and ixazomib are emerging. Various mechanisms of action of proteasome inhibitors were implicated in ALL. The expression of immunoproteasome is increased in ALL and thus selective inhibition of immunoproteasome is a promising approach in achieving maximal therapeutic efficacy and minimizing undesirable side effects.

However, large-scale clinical trials of proteasome inhibitors in treating relapse/refractory adult ALL are lacking. Moreover, the use of proteasome inhibitors-based regimen in treating newly diagnosed ALL is under-investigated. Further clinical studies are needed to establish the role of proteasome inhibitors in treating newly diagnosed ALL and relapse/refractory ALL in adult. Moreover, clinical trials of newer generation of proteasome inhibitors are also needed.

Despites its therapeutic efficacy, the mechanism of action of proteasome inhibitors are not well understood in certain subtypes of ALL, particularly *NOTCH1* wild-type T-ALL including ETP-ALL and Ph-like ALL which confer a poor prognosis (35, 77, 78). Further studies are needed to elucidate the underlying mechanism of action of proteasome inhibitors in those subtypes of ALL. Optimal combination therapeutic strategy can be developed after throughout understanding the mechanism of action. It can enhance the therapeutic efficacy of proteasome inhibitors and thus improve the prognosis of patients with ALL. Lastly, the mechanism of action of newer proteasome inhibitors is under-investigated and more mechanistic studies are needed.

## Author Contributions

CS planned and conceptualized the article. Both CS and MM wrote the article. The article had been reviewed by both CS and MM before submission.

## Conflict of Interest

The authors declare that the research was conducted in the absence of any commercial or financial relationships that could be construed as a potential conflict of interest.

## Publisher’s Note

All claims expressed in this article are solely those of the authors and do not necessarily represent those of their affiliated organizations, or those of the publisher, the editors and the reviewers. Any product that may be evaluated in this article, or claim that may be made by its manufacturer, is not guaranteed or endorsed by the publisher.
